# How We Do Harm: Do Copyrighted Scales Benefit Research in the Developing World?

**DOI:** 10.3389/fpubh.2019.00377

**Published:** 2019-12-18

**Authors:** Md. Dilshad Manzar, Mohammed Salahuddin, Muktar Sano Kedir, Vijay Kumar Chattu, David Warren Spence, Seithikurippu R. Pandi-Perumal

**Affiliations:** ^1^Department of Nursing, College of Applied Medical Sciences, Majmaah University, Al Majma'ah, Saudi Arabia; ^2^Department of Pharmacy, College of Medicine and Health Sciences, Mizan-Tepi University, Mizan-Aman, Ethiopia; ^3^Department of Paraclinical Sciences, Faculty of Medical Sciences, The University of the West Indies, St. Augustine, Trinidad and Tobago; ^4^Department of Public Health Research, Global Institute of Public Health, Thiruvananthapuram, India; ^5^Independent Researcher, Toronto, ON, Canada; ^6^Somnogen Canada Inc., Toronto, ON, Canada

**Keywords:** cost analysis, health equity, health inequality, health policy, global health promotion, health research policy

## Abstract

One of the most difficult challenges in carrying out global health research in the developing world is the issue of copyright protection of questionnaires. The current reality is that research in the developing world is often hampered by inadequate or even non-existent budgetary support. From our point of view, an additional hindrance to carrying out research in developing countries is the insistence by holders of questionnaire copyrights that they are paid for the use of their testing instruments. One adverse consequence of demands for compensation by copyright holders may be that worthwhile research is impeded or even prevented. It is argued that the practice of charging non-funded research projects for the use of copyrighted questionnaires denies inclusion of data on world minorities, and thus prevents the potential benefits that such data could provide. In this commentary, we focus on copyrighted instruments and the restrictions that they often represent for researchers in the developing world. More broadly, we argue that to the extent that research in the developing world is impeded by demands for developed world levels of compensation for the use of proprietary tests, the development of vital health programs that are designed to serve these populations can be adversely affected. Several strategies for rectifying inequities posed by current copyright policies are suggested for the promotion of health research in the developing world.

It has been noted that the development of healthcare programs, and ultimately the achievement of the goal of universal health, are critically dependent on healthcare research (1). Historically however the priorities of global health research have been largely determined by decision makers in the developed world, i.e., those who have the financial resources to underwrite such research (2). These realities often misrepresent the actual public health problems occurring in the developing world, problems which are usually best understood by local researchers who are closer to the public health difficulties of their home environments. A frequently encountered problem is that a mismatch occurs between the amount of research that is carried out in the developing world and the severity of healthcare needs in these areas. Many diseases that have been eradicated in the developed countries are still endemic across much of the developing world and continue to exist only among the world's poorest and most marginalized communities, where it affects the most vulnerable in local populations. Mendis et al., carried out a literature survey of 3000 references and found that, while 75% of the worldwide incidence of cardiovascular disease is concentrated in developing countries, a disproportionately small amount of research was actually being conducted there. They found that 78% of research came from developed countries, but that only 6–8% was from developing ones (3). African researchers contributed only 1% to global clinical medical publications between 2004 and 2008, nearly half of which was carried out by those based in South Africa (4). It has also been found that <10% of global funding for research is spent on diseases that afflict more than 90% of the world's population (5). Taken together these findings support the inference that public health in the developing world is closely linked to the health research that is carried out in these countries, and, by extension, those factors that limit health research will ultimately have effects on the health of local populations.

Analyses of the reasons for the lack of research in the developing world nearly always point to a lack of adequate research funding (6). This state of affairs affects research at both the program level and in terms of attracting qualified personnel, with surveys showing that young scientists in these countries often do not view research as a viable career option, largely because of the severe limitations in financial support (6–8). An additional challenge to carrying research in the developing world is the existence of many basic expenses, which in research projects in developed countries are accepted as routine budget requirements. Often the significance of these expenses goes unrecognized by suppliers of research equipment or tools, which unfortunately may affect both the planning and carrying out of research studies. It has been suggested that greater collaborative research efforts could be fostered if there were a greater understanding of both the potential and difficulties of research in poor countries (9). Among these difficulties are the restrictions that are imposed by copyrighted questionnaires.

Over the years, our own research group has used a number of scales and questionnaires. In many cases, we were able to obtain consent, permission, and copyright clearance directly from the authors. On other occasions, the use of specific copyrighted questionnaires would have entailed substantial charges. In several instances of doing international research, we found that the developers of survey instruments had transferred the copyrights to private healthcare companies. Challenges occurred especially when we had to pay attention to copyright issues prior to the design of our studies. Unfortunately, financially motivated developers of questionnaires, as well as some institutions and companies, frequently demand substantial fees for the use of these instruments, thus putting them beyond the reach of many researchers in low-income countries in the world where, due to economic constraints, research must be carried out with little or no funding (10). In a recent survey, it was found that lack of funding for research as well as for research equipment were the most frequently cited complaints by young scientists in Africa when asked what they viewed as the main challenges to doing research in their countries (11).

In more than one instance, we dealt with the problem of copyright fees, and prior to a study needed to determine whether a particular scale could be used. Many of the studies that we carried out in Ethiopia were not formally funded by either a government or private entity. In fact, out of a passion for research, the enthusiastic researchers used portions of their salaries to create a pool of money. Despite this dedicated commitment, the investigators' research goals sometimes had to be modified because of unwillingness on the part of copyright holders to work with the extremely limited budgets that tend to be the norm of many research projects in the developing world.

In one case, one of our Ethiopian collaborators was asked to visit a seminar in a European city to obtain a free license for a questionnaire. Another problematic instance involved our attempt to gain access to the Headache Impact Test (HIT-6), which we discussed with a series of people from QualityMetric, now part of Optum (https://www.optum.com). After a lengthy email discussion in which we provided all the information requested, Optum eventually turned down our request and insisted that their surveys and the scoring software be paid for.

Such experiences suggest that there are often serious inequities in the way charges are applied to survey research in the developing world, which ultimately affects the inclusiveness of the research. We believe that relevant to this point are the findings of Wendler et al., which were used to support the authors' conclusions that the non-inclusion of minority respondents in survey research is not due to resistance or unwillingness of such populations to participate, but rather to flaws in the structuring of research which restricts access (12). We are also reminded of a view expressed by Spong and Bianchi (13), i.e., that many underrepresented populations encounter barriers to participation in survey or health research, often due to implicit policies in the recruitment process that are adverse to their inclusion, or due to failure by investigators to even recognize the existence of certain subpopulations. Further, to determine the extent of inequity in published research based on copyrighted questionnaires, we randomly chose three copyrighted questionnaire tools from eprovide (https://eprovide.mapi-trust.org/) and ascertained their use in published research (based on citations as on 11.11.2019) in developing countries as well as in developed regions of the world (Table 1). More than 91% of the articles that cited these three questionnaires were published in developed countries (Table 1) (14–16). The net effect of these practices is to prevent the gathering of information that would ultimately help more people. It is our opinion that such intransigent attitudes among copyright holders are contrary to the ethos and spirit of the promotion of science in underdeveloped regions. Our repeated experience has been that the defenders of such attitudes toward copyright are entirely oblivious to the realities of research in the developing world. What is the practical solution to this problem? We briefly discuss three strategies as a way ahead.

**Table 1:** Some copyrighted questionnaire tools and their citations in published research.

**Serial number**	**Questionnaire**	**Total citations as on 11.11.2019**	**Number of citing articles published in developing and underdeveloped countries**	**Number of citing articles published in developed countries**
1	Haemo-SYM (Haemo-SYM)	5	0	5
2	Peyronie's Disease Questionnaire (PDQ)	100	5	95
3	Osteoporosis Patient Satisfaction Questionnaire (OPSAT-Q)	47	8	39

## Researchers in the Developing Countries: More Persuasive and Informed Approach

Researchers can consider taking the following course of action: During the conception phase of their project, they should carefully evaluate each scale or questionnaire and its copyright issues, querying the group or individual copyright holder to obtain permission. The developer might respond that a private firm holds the copyright. If the firm unambiguously provides free licensing for non-funded projects, then researchers from the developing world can utilize the instrument. As an initial step in the process of realizing this objective, especially among those who must contend with the practical need to carry out research in the immediate present, it is further recommended that developing country researchers contact questionnaire copyright holders and explain their specific circumstances. Additionally, they should include copies of various articles that have dealt with this issue, including a list of references, in their request for justified consideration.

Otherwise, to prevent legal issues, we suggest avoiding the use of such questionnaires. Among several published examples illustrating this problem is the well-documented case of Donald E. Morisky, Research Professor of Jonathan and Karin Fielding School of Public Health, University of California, Los Angeles, USA (17–23). In this instance, Morisky denied a researcher permission to use his scale for measuring medication adherence unless a substantial fee were paid in advance. The researcher then rewrote the scale by changing the phrasing of individual questions, although the essential topics of the scale remained close to the original. The researcher used the modified scale and later published his results. Morisky successfully sued the researcher for copyright violations.

In such circumstances, researchers should try to identify potential alternative scales and questionnaires. There may be other similar questionnaires that might be closer to the one that they intended to use. The other option, though not tenable everywhere, would be for researchers to develop their own, alternative versions of desired scales. When researchers employ these alternatives, the reliance on copyrighted tools will eventually diminish. This may also eventually result in a low article citation of the copyrighted instrument, which will have serious implications for the h-index, a bibliometric indicator of the individual academic scholarship of the authors of the protected instrument. An h-index is an indicator of an author-level metric, which measures academic productivity both quantitatively and qualitatively evaluate an individual's scholarly output (24, 25). The H-index is the most common publication statistic that is designed to assess the data obtained from past achievement and is often used to predict promotion worthiness in academia. It thereby provides a useful metric for scientists who wish to summarize their research accomplishments.

## Research Funders and Publishers: Proactive Role in the Development of Policy for Data Sharing Specially for Inclusive Research Growth

It is our opinion that publishers and journals can facilitate research in the developing world by creating policies and consensus statements concerning data sharing. In such cases, if they happen to publish a new instrument, the future course of action needs to be established. Many journals have clear open access policies for article processing charges (APC). However, clear policies regarding data sharing by researchers are still lacking. For example, when publishing a scale or a questionnaire, the publishers should clarify if the authors intend to patent their findings or to apply for potential licenses or copyrights for their scales and questionnaires. Further, whether the copyright will be held individually or institutionally should be specified clearly, as well as any intention to transfer copyrights to other for-profit companies. It is frequently the case that publishers and journals fail to identify the potential financial conflicts of interest concerning questionnaire developers. Some might not even disclose their economic ties with the companies to which they have transferred the copyright. With regard to the funding agencies and the grantee, the copyright and intellectual property issues must be clarified. A critical question relates to whether questionnaire developers could copyright their own intellectual products if public funds had been used to sponsor their project. In this circumstance, the question would be to evaluate who is the legally liable party, the questionnaire developer, the grantee, or both. We argue that, as global health stakeholders, we need to overcome the barriers to carrying out scientific research and reduce the gap in the research output between developed and developing nations. Globalization and opportunities for growth have not resolved the disparities that exist between developed and developing nations and there is an urgent need to close this gap and encourage research in these countries where it is most needed. Developing nations, which make up a major percentage of the world's population, also contribute significantly to the global burden of disease. According to the World Health Organization, developing regions carry a disproportionately heavy share of the environmental disease burden when compared to that of developed countries (26). It is thus essential that the basic needs of these populations be addressed through the support of basic health research.

## Questionnaire Developers: Adoption of Global Attitude Adjustments

We feel that help must come from questionnaire developers, institutions, and industries at large. We take the position that recognition of the economic challenges, which face researchers in developing countries, will ultimately benefit the populations of these areas by promoting basic standards in public health. More specifically, we believe that support of inclusive health agendas in developing countries should take priority over the needs of questionnaire developers to protect their intellectual property. It is our view that the developed world should take meaningful actions toward connecting with researchers in the less developed world. This should include efforts to support health research within their countries and also to provide external support when and where it is needed (27).

To a great extent, the resolution of the disparity between the perceptions of developed world questionnaire developers and researchers in poor countries is one of advocacy and education. It would be comforting to believe that that this objective could be achieved through one or two international conferences on cultural sensitivity or some analog such as “sensitivity to research needs in the developing world.” However, this message will have to be continuously repeated over many years. Culturally based perceptions, one might even say misperceptions, are frequently entrenched and represent “a tough nut to crack.” The overcoming of cultural myopias is always challenging and requires education. There is an additional need for carefully planned opportunities for those with differing views to work together and to appreciate unfamiliar perspectives on problems. This process does not happen automatically and requires carefully planned programs of attitude modification. Advocacy through editorials in research journals is one strategy (28). Encouragement of collaborative international research projects in another. A further strategy would be sponsorship of research into, e.g., questionnaire development by their original developed world creators. This could be incentivized by encouraging recognition that these intellectual products would gain international exposure and thus, an elevated scientific recognition worldwide. As a consequence, the participation of local scientists would be necessitated.

In summary, sustained efforts involving suggested measures of: (i) a more persuasive and informed approach by researchers in the developing countries, (ii) proactive development of policy for data sharing by research funders and publishers, and (iii) adoption of global attitude adjustments by questionnaire developers in the developed countries may help remove disparity in the health research. It is to be hoped that persistent efforts in this regard will foster a more inclusive perspective on the needs of researchers in the developing world.

## Author Contributions

MM, MS, MK, and SP-P: conceptualized, designed, and implementation of the manuscript and drafted the paper. VC and DW: provided materials and contextual information, advised the studies inclusion, and critically reviewed, revised, and finalized the manuscript. All authors met the ICMJE criteria for authorship and approved the final version of the submitted manuscript.

### Conflict of Interest

SP-P is a stockholder and the President and Chief Executive Officer of Somnogen Canada Inc., a Canadian Corporation. He declared no competing interests that might be perceived to influence the content of this article. The remaining authors declare that the research was conducted in the absence of any commercial or financial relationships that could be construed as a potential conflict of interest.
